# PCSK9 Antibodies for the Treatment of Hypercholesterolemia

**DOI:** 10.3390/nu6125517

**Published:** 2014-12-01

**Authors:** Ioanna Gouni-Berthold, Heiner K. Berthold

**Affiliations:** 1 Center of Endocrinology, Diabetes and Preventive Medicine (ZEDP), University of Cologne, Kerpener Str. 62, Cologne 50937, Germany; 2 Department of Internal Medicine and Geriatrics, Bielefeld Evangelical Hospital (EvKB), Schildescher Str. 99, Bielefeld 33611, Germany; E-Mail: heiner.berthold@evkb.de

**Keywords:** PCSK9, PCSK9 antibodies, evolocumab, alirocumab, low-density lipoprotein cholesterol

## Abstract

The serine protease proprotein convertase subtilisin/kexin type 9 (PCSK9) binds to the low-density lipoprotein (LDL) receptor (LDLR) and directs it to lysosomes for intracellular degradation. This results in decreased numbers of LDLR available on the hepatic cell surface to bind LDL particles and remove them from the circulation and therefore to a subsequent increase in circulating LDL-cholesterol (LDL-C) plasma levels. Since 2003, when the role of PCSK9 in LDL-C metabolism was discovered, there have been major efforts to develop efficient and safe methods to inhibit it. Amongst those, monoclonal antibodies against PCSK9 are the furthest in development, with multiple phase 3 trials already published and with cardiovascular endpoint trials currently underway. Two fully human monoclonal antibodies, evolocumab (AMG 145) and alirocumab (REGN727/SAR236553), have been extensively studied in a wide range of subjects, such as those with statin intolerance, as an add-on to statin therapy, as a monotherapy and in patients with familial hypercholesterolemia. PCSK9 antibodies result in a consistent and robust decrease in LDL-C plasma levels ranging from 40% to 70%, either on top of statins or as a monotherapy. If the safety data from the on-going phase 3 trials remain as reassuring as the data available till now, PCSK9 antibodies will offer a novel, powerful therapeutic option to decrease LDL-C plasma levels and, hopefully, cardiovascular risk.

## 1. Introduction

Proprotein convertase subtilisin kexin type 9 (PCSK9) was initially identified in 2003 as neural apoptosis-regulated convertase 1 (NARC-1), a protein that is upregulated in primary cerebellar neurons that undergo apoptosis induced by serum deprivation [1]. At the same time, Abifadel *et al*. [2] described two gain-of-function mutations in the PCSK9 gene causing autosomal dominant hypercholesterolemia in two French families without mutations in the typical candidate genes responsible for familial hypercholesterolemia, the low-density lipoprotein (LDL) receptor (LDLR) and apolipoprotein B (apoB). Three years later, loss-of-function mutations in the PCSK9 gene were found to be associated with low LDL-cholesterol (LDL-C) levels and reduced cardiovascular risk [3]. These observations led to intensive research to identify the mechanism(s) through which PCSK9 regulates LDL-C levels.

PCSK9 is the ninth member of the proprotein convertase superfamily, a 692-aminoacid glycoprotein expressed mainly in the liver, but also in the small intestine, kidney and the central nervous system [4]. PCSK9 is synthesized as a pro-protein, proPCSK9, having a signal peptide (amino acids 1–30), a prodomain (amino acids 31–152), a catalytic domain, a hinge domain and a Cys-His-rich domain (CHRD) [5]. ProPCSK9 catalyzes itself in the endoplasmic reticulum, but the prodomain remains attached to the catalytic site, blocking any further proteolytic activity. Thus, the only substrate so far described for PCSK9 is itself. After PCSK9 is secreted from the cell, it binds the epidermal growth factor-like repeat A (EGF-A) domain of the LDLR on the liver surface and guides it for degradation in the lysosomes. Therefore, the physiological recycling of the LDLR to the liver surface is cancelled, less LDLR is present on the liver surface and, as a result, the circulating LDL-C plasma levels rise [5].

A number of factors modulate circulating PCSK9 concentrations, such as the fasting state (associated with a decrease in PCSK9 levels), gender (PCSK9 concentrations are higher in women than in men) and the time of the day (nadir concentrations are between 3 pm and 9 pm and peak at around 4 am) [6]. Interestingly, PCSK9 and the LDLR are transcriptionally co-upregulated via the sterol regulatory element binding protein-2 (SREBP2) pathway after treatment with statins. Thus, statin treatment is associated with an increase in circulating PCSK9 levels, as well as with increased numbers of LDLR, a phenomenon which could, at least partially, explain the “rule of six”, namely the observation that doubling a statin dose results in only a ~6 percentage points further decrease in LDL-C plasma levels [7]. On the other hand, the cholesterol absorption inhibitor, ezetimibe, does not increase circulating PCSK9 concentrations despite its cholesterol-lowering effects [8].

Since loss-of-function mutations of the PCSK9 gene were found to be associated with a 28% reduction in LDL-C levels and an 88% reduction in the risk of coronary heart disease (CHD) in black individuals and with a 15% reduction in LDL-C and a 47% risk reduction in whites [3], an intense effort to develop the optimal method for blocking the action of PCSK9, especially from the part of the pharmaceutical industry, was initiated. Various approaches were tried, such as antisense oligonucleotides blocking PCSK9 synthesis (discontinued development in 2011) [9] and small interfering RNA targeting PCSK9 synthesis (results of a phase 1 trial showed a 40% reduction of LDL-C compared to placebo after intravenous infusion in healthy volunteers) [10]. Another approach that has been tried, but recently abandoned [11], was inhibitory adnectins specific for PCSK9. They comprise a scaffold of the tenth extracellular human fibronectin type III domain that exposes a PCSK9-binding loop [5]. Smallmolecules inhibiting the interaction between PCSK9 and the LDLR are still in pre-clinical development, and the relative flatness of the surface of interaction between PCSK9 and the LDLR may make their development quite challenging [5,11]. In this article, we are going to present the approach that is the most advanced in clinical development, the PCSK9 antibodies, and, specifically, the published results of phase 3 trials with evolocumab (AMG 145) and alirocumab (REGN727/SAR236553). We refrain from presenting results that have been published only as abstracts, since discrepancies between abstract results and subsequent full-length publication results are quite common [12].

Currently, there are three PCSK9 antibodies undergoing phase 3 trials. Two fully human antibodies, evolocumab from Amgen, alirocumab from Sanofi/Regeneron and one humanized antibody, bococizumab (RN316/PF-04950615) from Pfizer. The results of the published phase 2 trials with evolocumab and alirocumab, namely four with evolocumab (as a monotherapy [13], as a combination therapy [14], in patients with heterozygous familial hypercholesterolemia (heFH) [15] and in statin-intolerant patients [16], as well as an open-label 52 week trial [17]) and three with alirocumab (as a combination therapy [18,19] and in patients with heFH [20]) have been recently thoroughly reviewed [6,21].

### 1.1. Lipid Effects

In the phase 2 trials of evolocumab and alirocumab, there were mean reductions of LDL-C from baseline ranging from 40% to 72% (usually 50%–60%) when combined with a statin or as a monotherapy [6,22]. Moreover, they were shown to decrease apoB, total cholesterol (TC) and non-high-density lipoprotein cholesterol (non-HDL-C) concentrations to a degree similar to that of LDL-C [21]. Modest increases in HDL-C and decreases in triglycerides have been reported in almost all trials. Interestingly, in all trials, a reduction in lipoprotein(a) (Lp(a)) concentrations consistently ranging between 15%–30% was observed. The mechanism mediating this unexpected effect remains unclear [23,24].

### 1.2. General Safety

The safety profile of these drugs based on the currently available evidence seems to be very good. The rates of adverse events of both antibodies in phase 1 and 2 trials have been comparable to placebo [7]. The potential for antibody development against evolocumab and alirocumab has been rigorously monitored. Binding antibodies were observed in one patient each in the evolocumab and placebo groups [13], while no neutralizing antibodies were detected in any of the evolocumabtrials [13,14,15,16]. In alirocumab trials, antibodies against the drug were observed in eight patients at minimally detectable levels [18,19]. The rates of muscle-related adverse events, elevated creatine kinase (CK) or aminotransferases greater than 5- to 10-times the upper limit of normal (ULN) were not significantly different in patients treated with evolocumab or alirocumab compared with controls [7].

Taken together, the published evidence from the phase 2 trials suggests that PCSK9 inhibition with monoclonal antibodies is an effective and well tolerated treatment option for a diverse patient populationswith hypercholesterolemia over a 12-week treatment period. Results from phase 2 trials with bococizumab have only been presented till now as abstracts, and no results are available on the phase 3 trials.

## 2. Phase 3 Trials with Alirocumab

The ODYSSEY phase 3 clinical trial program has been designed to further assess the efficacy and safety of alirocumab. The program consists of 14 trials, including more than 23,500 patients and over 2000 study centersworldwide, and evaluates alirocumab in various clinical scenarios, such as in combination with statins, as a monotherapy in a statin intolerant population (ODYSSEY ALTERNATIVE), as well as the effects of alirocumab in addition to statin therapy in a large cardiovascular outcomes trial (ODYSSEY OUTCOMES). ODYSSEY Mono is the first published report from the ODYSSEY program.

### ODYSSEY Mono

Roth *et al.* [25] performed a phase 3, randomized, double-blind, double-dummy study in patients with LDL-C of 100–190 mg/dL, which had a moderate cardiovascular risk (10-year risk of fatal cardiovascular events ≥1% and <5% based on the European Systematic Coronary Risk Evaluation [26]), to compare the efficacy and safety of alirocumab with ezetimibe. The subjects were not receiving statins or any other lipid-lowering therapy and were randomized to ezetimibe 10 mg/day (*N* = 51) or alirocumab 75 mg subcutaneously (SC via a 1-mL autoinjector every two weeks (Q2W) (*N* = 52), with the dose up-titrated to 150 mg Q2W (also 1 mL) at Week 12 if by Week 8 LDL-C was still ≥70 mg/dL. The majority of patients chose to self-administer the injections. Ezetimibe was selected as the comparator to alirocumab, since it is the most common treatment option used in patients with statin intolerance [27]. The trial used a previously unstudied alirocumab dose, 75 mg every two weeks (Q2W), which was chosen based on modelling data from the alirocumab phase 2 trials. The primary endpoint was the mean LDL-C percent change from baseline to 24 weeks, analyzed using an intention-to-treat approach (ITT). Analyses using on-treatment LDL-C values were also performed. Mean ± SD baseline LDL-C levels were 141 ± 27 mg/dL with alirocumab and 138 ± 25 mg/dL with ezetimibe. The 24-week treatment period was completed by 85% of alirocumab and 86% of ezetimibe patients. LDL-C reductions were 47% with alirocumab *vs.* 16% with ezetimibe (*p* < 0.0001 using ITT analysis) and 54% *vs.* 17% (*p* < 0.0001 using on-treatment analyses). At Week 12, before up-titration, alirocumab 75 mg Q2W reduced LDL-C by 53% (on-treatment analysis). Percent reductions from baseline in apoB (36.7% *vs.* 11.0%), TC (29.6% *vs.* 10.9%) and non-HDL-C (40.6% *vs.* 15.1%) were significantly greater for alirocumab *vs.* ezetimibe at Week 24 and similar in the ITT and on-treatment analyses. Moderate reductions in Lp(a) and triglycerides and increases in HDL-C were observed following both of the study treatments, with no significant differences between the alirocumab and ezetimibe arms.

Safety: In the alirocumab arm, 69% of the patients experienced at least one adverse event (AE), and in the ezetimibe arm, 78%. There were no deaths. Two serious AEs (SAEs) were reported: one patient, who had received alirocumab 75 mg Q2W for three months and had a history of atrial fibrillation and chronic obstructive pulmonary disorder had a pulmonary embolism. Alirocumab was discontinued, and the patient was hospitalized and subsequently recovered. One patient in the ezetimibe arm with a medical history of arthritis experienced glenoid erosion and was hospitalized for shoulder arthroplasty. The patient recovered in hospital and completed the study. Neither of the SAEs were considered by the investigator to be related to the study treatment. Treatment-emergent AEs occurring in 5% or more patients in the alirocumab and ezetimibe treatment arms were, respectively, nasopharyngitis 23.1% *vs.* 15.7%, diarrhea 11.5% *vs.* 3.9%, influenza 11.5% *vs.* 5.9%, arthralgia 5.8% *vs.* 3.9%, nausea 3.8% *vs.* 9.8%, back pain 1.9% *vs.* 5.9%, dizziness 1.9% *vs.* 5.9% and urinary tract infection 0% *vs.* 5.9%. Nine patients prematurely discontinued study treatment following one or more treatment-emergent AE (10% of patients in the alirocumab arm and 8% in the ezetimibe arm). In the alirocumab group, treatment-emergent AEs leading to discontinuation were pulmonary embolism (*N* = 1), nausea, fatigue, headache and flushing (*N* = 1), generalized aching (*N* = 1), injection site reaction (*N* = 1) and diarrhea (*N* = 1). In the ezetimibe group, the treatment-emergent AEs leading to discontinuation were gout (*N* = 1), fatigue, back pain and frequent urination (*N* = 1), abdominal cramping and injection site reaction (*N* = 1) and vivid dreams (*N* = 1).

Muscle-related treatment-emergent AEs occurred in 4% of alirocumab patients and in 4% of the patients receiving ezetimibe. Elevated CK levels over 10-times the ULN were reported in one patient in the ezetimibe group. Two percent of the patients in the alirocumab group experienced a local injection site reaction and 4% in the ezetimibe group. Three patients who were treated with alirocumab 75 mg Q2W experienced at least one LDL-C value <25 mg/dL. Of note, in this study, LDL-C measurements were done using the Friedewald formula, which is not so precise at such low LDL-C levels. There were no increases over three-times the ULN in alanine aminotransferase or aspartate aminotransferase. Six patients had blood glucose ≥126 mg/dL in the alirocumab arm *vs.* one in the ezetimibe arm. However, the patients in the alirocumab arm who experienced high blood glucose during the treatment period had abnormal fasting glucose at screening or baseline, and three of them had also baseline HbA1c of more than 6.5%. There was no pattern observed in changes in either blood glucose or HbA1c from screening to Week 24. Treatment-emergent anti-drug antibodies were found in six patients in the alirocumab arm and in no one in the ezetimibe arm. Five of these patients were classified as having a persistent responserecorded at the follow-up visit. For all anti-drug antibody-positive patients, titers were low, and no neutralizing anti-drug antibodies, which may affect alirocumab pharmacokinetics, LDL-C effects or safety, were detected.

In summary, this study showed that alirocumab decreases LDL-C plasma levels significantly more compared to ezetimibe after 24 weeks of treatment with the lower 75 mg Q2W dose, which resulted in a ≥50% LDL-C reduction in the majority of patients. Adverse events were comparable between groups.

## 3. Phase 3 Trials with Evolocumab

### 3.1. The DESCARTES Trial (Durable Effect of PCSK9 Antibody Compared with Placebo Study)

Purpose of the DESCARTES phase 3 trial [28] was to evaluate the safety and efficacy of 52 weeks of treatment with evolocumab. Patients with hyperlipidemia were stratified according to the risk categories outlined by the Adult Treatment Panel III (ATP-III) of the National Cholesterol Education Program (NCEP). Based on this classification, patients were started on background lipid-lowering therapy with diet alone or diet plus atorvastatin 10 mg daily or 80 mg daily, or atorvastatin 80 mg daily plus ezetimibe 10 mg daily, for a run-in period of 4 to 12 weeks. Patients with an LDL-C level ≥75 mg/dL were then randomly assigned in a 2:1 ratio to receive either evolocumab (420 mg) or placebo every four weeks. The primary end point was the percent change from baseline in LDL-C at Week 52. Nine hundred and one patients were included in the primary analysis. The overall least squares mean ± SE reduction in LDL-C from baseline in the evolocumab group, taking into account the change in the placebo group, was 57.0% ± 2.1% (*p* < 0.001). In patients who had a background therapy consisting of diet alone, the mean reduction was 55.7% ± 4.2%, in patients who received atorvastatin 10 mg 61.6% ± 2.6%, in patients who received atorvastatin 80 mg 56.8% ± 5.3% and in patients who received atorvastatin 80 mg and 10 mg ezetimibe 48.5% ± 5.2% (*p* < 0.001 for all comparisons). LDL-C levels were reduced below 70 mg/dL in 82.3% of patients in the evolocumab group, compared with 6.4% in the placebo group. Evolocumab also significantly decreased the concentrations of apoB (44.2%), non-HDL-C (50.3%), Lp(a) (22.4%) and triglycerides (11.5%). HDL-C was increased significantly by 5.4%.

Safety: The overall incidence of adverse events occurring during treatment was similar in the evolocumab group and the placebo group, with 74.8% and 74.2%, respectively, having an adverse event. The most common adverse events in the evolocumab group were nasopharyngitis, upper respiratory tract infection, influenza and back pain. Serious adverse events occurred in 33 patients (5.5%) in the evolocumab group and in 13 patients (4.3%) in the placebo group. Adverse events leading to the discontinuation of a study drug occurred in 2.2% in the evolocumab group and in 1.0% in the placebo group. Injection-site reactions were reported in 5.7% in the evolocumab group and in 5.0% in the placebo group. One patient in the evolocumab group discontinued treatment due to this side effect. Elevations of CK levels to more than five-times the ULN occurred in 1.2% of patients in the evolocumab group and 0.3% of the patients receiving placebo. Myalgia was reported in 4.0% of the patients in the evolocumab group and in 3.0% of the patients in the placebo group. Elevations of aminotransferase levels to more than three-times the ULN occurred in 0.8% and 1.0% in the evolocumab and placebo groups, respectively. Glycemic indexes were not altered by evolocumab treatment. Two patients in the evolocumab group had detectable binding antibodies before or at the time of randomization. One patient in the evolocumab group had transient anti-evolocumab binding antibodies during treatment. No anti-evolocumab neutralizing antibodies were detected in any patient.

In summary, this study showed that evolocumab added to diet alone, or to low-dose atorvastatin, or to high-dose atorvastatin with or without ezetimibe significantly reduces LDL-C levels in patients with a range of cardiovascular risks over a period of 52 weeks.

### 3.2. The LAPLACE-2 Trial (LDL-C Assessment with PCSK9 Monoclonal Antibody Inhibition Combined with Statin Therapy)

The purpose of the 12-week, randomized, double-blind, placebo- and ezetimibe-controlled LAPLACE-2 trial [29] was to evaluate the efficacy and tolerability of evolocumab when used in combination with a moderate- *vs.* high-intensity statin in patients with primary hypercholesterolemia and mixed dyslipidemia. A total of 2067 patients were randomized to a daily, moderate-intensity statin, defined as atorvastatin 10 mg, or simvastatin 40 mg, or rosuvastatin 5 mg, or to high-intensity statin, defined as atorvastatin 80 mg or rosuvastatin 40 mg. After a four-week lipid-stabilization period, a total of 1899 patients were randomized to compare evolocumab (140 mg SC every two weeks or 420 mg SC monthly) with (i) placebo SC (every two weeks or monthly) or (ii) ezetimibe (10 mg or placebo daily only for patients receiving atorvastatin) when added to statin therapies. The main outcome was the percent change from baseline in LDL-C plasma levels at the mean of Weeks 10 and 12 and at Week 12. Evolocumab reduced LDL-C levels by 66%–75% when given every two weeks and by 63%–75% when given monthly *vs.* placebo at the mean of Weeks 10 and 12 in the moderate- and high-intensity statin-treated groups. The LDL-C reductions at Week 12 were comparable. For moderate-intensity statin groups, evolocumab every two weeks reduced LDL-C from 115–124 mg/dL (baseline mean) to 39–49 mg/dL (on-treatment mean). Evolocumab once a month reduced LDL-C from 123–126 mg/dL (baseline mean) to 43–48 mg/dL (on-treatment mean). For high-intensity statin groups, evolocumab every two weeks reduced LDL-C from 89–94 mg/dL (baseline mean) to 35–38 mg/dL (on-treatment mean). Evolocumab once a month reduced LDL-C from 89–94 mg/dL (baseline mean) to 33–35 mg/dL (on-treatment mean). Evolocumab administered every two weeks and monthly resulted in significant reductions in non-HDL-C, apoB and Lp(a) concentrations in all groups. Specifically, evolocumab every two weeks reduced at the mean of Weeks 10 and 12 the non-HDL-C concentrations by 58%–65%, apoB by 51%–59% and Lp(a) by 21%–36% (all *vs.* placebo). The reductions in non-HDL-C, apoB and Lp(a) seen with evolocumab once a month were comparable to the every-two-weeks dosing. At the mean of Weeks 10 and 12, triglyceride concentrations were decreased by 12%–23% in patients receiving evolocumab every two weeks and by 14%–30% in patients receiving evolocumab monthly (all *vs.* placebo). HDL-C concentrations were increased by 4%–10% *vs.* placebo at the mean of Weeks 10 and 12 in both the every-two-weeks and the monthly dose.

Safety: Adverse events occurred in 36% of evolocumab-treated patients, 40% of ezetimibe-treated patients and 39% of placebo-treated patients. The most common adverse events in the evolocumab-treated patients were back pain, arthralgia, headache, muscle spasms and pain in the extremities (all <2%). From those five, musculoskeletal symptoms or headache were the most common adverse events. Adverse events resulting in study drug discontinuation were 1.9%, 1.8% and 2.2% in the evolocumab, ezetimibe and placebo groups, respectively. Serious adverse events were reported in 2.1% of evolocumab-treated patients, 0.9% of ezetimibe-treated patients and 2.3% of placebo-treated patients. Elevations in aspartate aminotransferase/alanine aminotransferase levels >3-times the ULN and CK elevations >5-times the ULN were uncommon in all groups. One death was reported during the study in a patient receiving rosuvastatin and subcutaneous placebo. Neurocognitive adverse events were reported in 0.1% of patients treated with evolocumab, 1.4% of patients treated with ezetimibe and in no patient treated with placebo. Injection site reactions were reported in 1.3% of evolocumab-treated patients, 0.9% of ezetimibe-treated patients and 1.4% of placebo-treated patients. Binding antibodies were detected in three patients treated with evolocumab prior to study drug administration. Of these, only one had detectable binding antibodies at the end of the study, and no new cases of binding antibodies after treatment were reported. Neutralizing antibodies were not detected.

In summary, this study showed that in patients with primary hypercholesterolemia and mixed dyslipidemia, evolocumab added to moderate- or high-intensity statin therapy results in additional LDL-C lowering.

### 3.3. The GAUSS-2 Trial (Goal Achievement after Utilizing an Anti-PCSK9 Antibody in Statin Intolerant Subjects)

The purpose of the GAUSS-2 trial [30] was to evaluate the efficacy and safety of evolocumab compared with oral ezetimibe in patients with hypercholesterolemia unable to tolerate effective statin doses (~10%–20% of patients receiving statins) [31]. The GAUSS-2 trial was a 12-week, double-blind study, which enrolled subjects 18–80 years of age on no statin or on a low-dose (weekly dose of seven-times the smallest available tablet strength) statin. Participants had LDL-C above their NCEP ATP-III risk category goal (≥100 mg/dL with diagnosed CHD or risk equivalent, ≥130 mg/dL without CHD or risk equivalent and ≥2 risk factors, ≥160 mg/dL without CHD or risk equivalent and one risk factor, or ≥190 mg/dL without CHD or risk equivalent and no risk factors). Participants had prior intolerance to ≥2 statins, defined as inability to tolerate any dose or increase the dose above the smallest tablet strength because of intolerable muscle-related side effects, which resolved or improved significantly upon dose decrease or discontinuation. They were randomized in a 2:2:1:1 ratio to the following arms: (i) evolocumab 140 mg SC every two weeks plus daily oral placebo, (ii) evolocumab 420 mg SC once monthly plus daily oral placebo, (iii) placebo SC every two weeks and oral daily ezetimibe 10 mg, or (iv) placebo SC monthly and oral daily ezetimibe 10 mg [32]. Co-primary endpoints were percent change from baseline in LDL-C at the mean of Weeks 10 and 12 and at Week 12. A total of 307 patients (age 62 ± 10 years, baseline LDL-C 193 ± 59 mg/dL) were randomized. Mean percent reductions from baseline at a mean of Weeks 10 and 12 were 56.1% with 140 mg every two weeks and 55.3% with 420 mg once monthly, corresponding to treatment differences *vs.* ezetimibe of 36.9% and 38.7%, respectively (*p* < 0.001), with similar mean percent reductions at Week 12 (*p* < 0.001). Evolocumab-treated patients were more likely to achieve LDL-C target levels than ezetimibe-treated patients (~76.5% *vs.* ~5.5%, respectively). Compared with ezetimibe, evolocumab resulted in significant reductions in apoB, Lp(a), non-HDL-C concentrations and in the apoB/apoA-I and TC/HDL-C ratios (*p* < 0.001).

Safety: Muscle-related AEs occurred in 12% of evolocumab-treated patients (13% in those treated every two weeks and 12% in those treated once monthly) and 23% of ezetimibe-treated patients. Treatment-emergent AEs and laboratory abnormalities were comparable across treatment groups.

AEs led to study drug discontinuation in 8% of patients on evolocumab and in 13% of patients on ezetimibe. Myalgia occurred in 8% of evolocumab-treated patients and 18% of ezetimibe-treated patients. Patients using low-dose statin therapy were more likely to develop myalgia both in the ezetimibe (statin *vs.* no statin: 21% *vs.* 17%) and the evolocumab group (statin *vs.* no statin: 17% *vs.* 6%). Discontinuation rates due to musculoskeletal side effects were 5% in the evolocumab and 6% in the ezetimibe groups, respectively. No binding or neutralizing antibodies to evolocumab were detected.

In summary, this study showed that evolocumab might be a promising therapy in high-risk patients with elevated LDL-C plasma levels who are statin intolerant. However, the study design did not include a blinded statin rechallenge, which is being addressed in the on-going GAUSS-3 (Goal Achievement after Utilizing an Anti-PCSK9 Antibody in Statin Intolerant Subjects) trial.

### 3.4. The MENDEL-2 Trial (Monoclonal Antibody against PCSK9 to Reduce Elevated LDL-C in Subjects Currently Not Receiving Drug Therapy for Easing Lipid Levels-2)

The purpose of the MENDEL-2 trial [33] was to compare evolocumab every two weeks or once a month with placebo and ezetimibe in patients with hypercholesterolemia. Patients 18–80 years of age with fasting LDL-C ≥100 and <190 mg/dL and Framingham risk scores ≤10% were randomized (1:1:1:1:2:2) to the following arms: (i) oral placebo and SC placebo every two weeks; (ii) oral placebo and SC placebo monthly; (iii) ezetimibe and SC placebo every two weeks; (iv) ezetimibe and SC placebo once a month; (v) oral placebo and evolocumab 140 mg every two weeks; or (vi) oral placebo and evolocumab 420 mg monthly. A total of 614 patients were randomized. Evolocumab treatment reduced LDL-C from baseline by 55%–57% more than placebo and 38%–40% more than ezetimibe(*p* < 0.001). Approximately 70% of the patients on evolocumab achieved a level of LDL-C <70 mg/dL compared to ~1.5% on ezetimibe and ~0.5% on placebo. Evolocumab significantly decreased also the levels of apoB, Lp(a) and non-HDL-C, as well as the ratios of TC to HDL-C and apoB to apolipoprotein A-I (apoA-I). HDL-C concentrations were significantly increased (*p* < 0.05). Triglyceride levels were significantly lowered with evolocumab once a month *versus* placebo or ezetimibe.

Safety: Treatment-emergent AEs, muscle-related AEs and laboratory abnormalities were comparable across treatment groups. Treatment-emergent AEs occurred in 44% of patients receiving evolocumab, 44% of the patients receiving placebo and in 46% of the patients receiving ezetimibe. No deaths or cardiovascular endpoints were reported. Serious AEs occurred in 1.3% of the patients receiving evolocumab *vs.* 0.6% in the placebo and 0.6% in the ezetimibe groups. In two cases, local investigators considered that events were related to the study drug. The first case was an acute pancreatitis in a patient on monthly evolocumab with a history of cholecystectomy, long-term alcohol intake and concomitant use of valproate (a drug known to induce pancreatitis [34]). The second case was a patient on evolocumab every two weeks who developed transaminase and CK levels eight-times the ULN (the values returned to normal after discontinuation of evolocumab). AEs led to study drug discontinuation in 3.9% of the patients in the placebo group, in 3.2% of the patients receiving ezetimibe and in 2.3% of the patients receiving evolocumab. Injection-site reactions were reported in 5% of each group, but none led to discontinuation of the study drug. No neutralizing or binding antibodies were detected duringthe trial.

In summary, this study, the largest monotherapy trial using a PCSK9 inhibitor to date, showed that evolocumab significantly reduces LDL-C plasma levels compared with placebo or ezetimibe and is well tolerated in patients with hypercholesterolemia.

### 3.5. The RUTHERFORD-2 Trial (The Reduction of LDL-C with PCSK9 Inhibition in Heterozygous Familial Hypercholesterolemia Disorder)

Previous studies with evolocumab in patients with heFH on stable lipid-lowering therapy have shown additional reductions in LDL-C of ~60% [15,28,35,36]. In the RUTHERFORD-2 trial [37] study, the relationship between LDL-C response to evolocumab and genotype was also investigated. The subjects, with an age ranging between 18 and 80 years, were defined as heFH when meeting clinical criteria for heFH. They were on stable lipid-lowering therapy for ≥4 weeks, with fasting LDL-C ≥100 mg/dL and were randomized 2:2:1:1 to receive SC evolocumab 140 mg biweekly, evolocumab 420 mg monthly, placebo biweekly or placebo monthly for 12 weeks. While the primary endpoint was the reduction in LDL-C, the response to evolocumab based on the mutations causing the disease was also evaluated. Of the 331 randomized patients, 264 agreed to the genetic analysis, and of those, 80% had mutations known to cause FH. For those found to have an LDL receptor mutation, patients were grouped by LDL receptor functional class (defective or negative) [38,39]. Patients with LDLR mutations that have been described to be causative of FH, but whose function has not yet been determined or described, were grouped as “unclassified”.

Evolocumab administered biweekly or monthly resulted in mean LDL-C reductions at Week 12 of 59% and 61%, respectively, *vs.* placebo (*p* < 0.001). Compared with placebo, LDL-C reductions with biweekly or monthly dosing were 61% and 55% in those with a LDLR mutation associated with no function, 49% and 66% in those with defective function, and 62% and 63% where the LDLR status was unclassified. Interestingly, reductions were variable even in patients with identical receptor mutations. Namely, in 13 out of 22 patients with the c.313 = 1G > A mutation randomized to evolocumab, the decreases in LDL-C at Week 12 ranged from 27% to 83%. The mean reductions at Week 12 in the seven patients who were either genetic homozygotes or compound heterozygotes were 68% with evolocumab 140 mg biweekly and 48% with 420 mg every month. In patients with apoB mutations, the reductions at Week 12 were 51% and 50%, with the biweekly and once monthly injections, respectively.

The placebo-adjusted mean reductions in apoB at Week 12 with evolocumab for the biweekly and monthly dosing groups, respectively, were 46% and 48% for patients with negative, 42% and 53% for patients with defective and 46% and 43% for patients with unclassified receptor status. Reductions in Lp(a) ranged from 19% to 45%, but did not appear to be dependent on the type of receptor mutation.

At Week 12, LDL-C <70 mg/dL was achieved by 68% and 63% of patients in the evolocumab140 mg biweekly and 420 monthly groups respectively, compared with 2% of patients in the placebo groups. Compared with placebo, mean reductions in Lp(a) were 32% and 28% at Week 12 (both doses *p* < 0.001).

Treatment with evolocumab 140 mg biweekly and 420 mg monthly compared with placebo resulted in mean decreases in triglycerides of 20% (*p* < 0.001) and 12% (*p* < 0.05), respectively, at Week 12. HDL-C was increased by 9% in both evolocumab groups.

Safety: The incidence of adverse events, positively adjudicated cardiovascular events, abnormal laboratory values, neurocognitive events and antibodies was comparable to rates reported in previous studies of evolocumab [15,35]. No SAEs led to study drug discontinuation, and no SAEs were considered related to evolocumab.

In summary, evolocumab given either 140 mg biweekly or 420 mg monthly resulted in reductions in LDL-C of ~60% in patients with heFH. Interestingly, the response to evolocumab was not related to the underlying genetic defect causing heFH.

### 3.6. TESLA Part B (Inhibition of PCSK9 with Evolocumab in Homozygous Familial Hypercholesterolemia (TESLA Part B): A Randomized, Double-Blind, Placebo-Controlled Trial)

Homozygous familial hypercholesterolemia (hoFH) is a rare disease caused by very low or absent plasma clearance of LDL, very high LDL-C plasma levels and the development of cardiovascular disease at a young age. The available pharmacotherapies achieve rather modest reductions of the LDL-C levels. In a pilot study of eight patients [40], evolocumab reduced LDL-C by 16%. In the TESLA Part B trial [41] study, 50 patients with hoFH on stable lipid-lowering therapy, and not on lipoprotein apheresis, received either subcutaneous evolocumab 420 mg or placebo every four weeks for 12 weeks. The primary endpoint was the percent change in LDL-C, measured with ultracentrifugation, from baseline at Week 12 compared with placebo. Of the 50 patients randomized, 49 completed the study (16 in the placebo group and 33 in the evolocumab group). Compared with placebo, evolocumab significantly reduced LDL-C by 30.9%. Treatment-emergent adverse events occurred in 63% of the patients in the placebo group and in 36% of the patients in the evolocumab group. No serious clinical or laboratory adverse events occurred, and no anti-evolocumab antibody development was detected during the study.

In summary, this study showed that in patients with hoFH receiving stable background lipid-lowering treatment and not on apheresis, evolocumab at a dose of 420 mg every four weeks significantly reduced LDL-C compared with placebo and was well-tolerated.

## 4. Authors’ Remarks

Considering the currently available data in its totality (Table 1), PCSK9 antibodies present a revolutionary therapeutic option that could benefit a wide-range of patients, such as those with familial hypercholesterolemia, patients with statin intolerance and patients at high or very high cardiovascular risk that cannot achieve their LDL-C target levels with the currently available lipid-lowering therapies. Interestingly, the muscle-related adverse events that can be seen in a number of patients treated with statins do not seem to occur with the use of PCSK9 antibodies. Moreover, all other lipid and lipoprotein fractions are modified advantageously with modest, but significant decreases in triglycerides and non-HDL-C, increases in HDL-C and an up to 30% decrease in Lp(a) concentrations. Due to the robust LDL-C lowering achieved with PCSK9 antibodies, levels of LDL-C well below 50 mg/dL will be observed in a number of patients treated with these agents. Although concerns about the safety of very low values of LDL-C had been raised over the years, focusing mainly on associations with cancer, violent death and hemorrhagic stroke, there has been very little evidence from outcomes trials to establish such a relationship [42].

**Table 1:** Efficacy of alirocumab (75 mg Q2W) and evolocumab (140 mg Q2W or 420 mg Q4W) on plasma lipids and lipoprotein concentrations: data from phase 3 clinical trials ^a^.

Trial	Study Description	LDL-C (%)	ApoB (%)	Non-HDL-C (%)	TG (%)	HDL-C (%)	Lp(a) (%)
*Alirocumab*							
ODYSSEY Mono [25]	Patients with hypercholesterolemia on no statins, compared to ezetimibe, 24 weeks ^b^	−31.6	−25.8	−25.5	−1.2	4.4	−4.4
*Evolocumab*							
DESCARTES [28]	Patients with hyperlipidemia 420 mg Q4W added to diet alone or to diet plus atorvastatin or to diet plus atorvastatin plus ezetimibe, 52 weeks	−57.0	−44.2	−50.3	−11.5	5.4	−22.4
LAPLACE-2 [29]	Patients with hypercholesterolemia, 140 mg Q2W or 420 mg Q4W added to moderate- or high-intensity statin therapy, compared to ezetimibe or placebo	−59.2, −70.6 *	−47.0, −61.4	−54.9, −66.6	−9.3, −31.4	3.2, 9.8	−19.8, −36.5
GAUSS-2 [33]	Patients with statin intolerance, 140 mg Q2W or 420 mg Q4W compared to ezetimibe ^b^	−68.8, −69.7	−32.9, −33.1	NA	NA	3.6, 4.8	−25.3, −27.9
MENDEL-2 [33]	Patients with hypercholesterolemia on no statins, 140 mg Q2W or 420 mg Q4W compared to ezetimibe	−54.8, −57.1	−47.8, −48.4	−49.8, −51.2	−6.2, −17.7	5.9, 9.3	−17.8, −20.4
RUTHERFORD-2 [37]	Patients with heFH, 140 mg Q2W or 420 mg Q4W	−59.2, −61.3	−49.1, −49.4	−54.8, 55.0	−11.6, −19.6	9.1, 9.2	−28.2, −31.6
TESLA Part B [38]	Patients with hoFH, not on apheresis, 420 mg Q4W	−30.9	−23.1	NA	0.3	−0.1	−11.8

TG, triglycerides; ApoB, apolipoprotein B; Q2W, every two weeks; Q4W, every four weeks; NA, not available. All studies are of a 12-week duration, except as indicated. ^a^ Results expressed as % treatment difference from baseline adjusted for placebo, except as indicated; ^b^ percent treatment difference *versus* ezetimibe; * observed range.

## 5. Conclusions

PCSK9 inhibition with fully human monoclonal antibodies appears to be a very promising method to robustly decrease LDL-C levels in various patient groups, such as those with or without FH, with or without statin intolerance and at various levels of cardiovascular risk. It is impressive that within just 10 years of the discovery of the role that PCSK9 plays in lipid metabolism, multiple phase 3 trials have been already completed and cardiovascular end-point trials are underway. While, till now, the safety profile of PCSK9 antibodies is excellent, long-term data and cardiovascular endpoint trials are needed before we can rather confidently attest to the safety and effectiveness of these compounds.
